# Urinary Mercury Levels in Children with Amalgam Fillings

**DOI:** 10.1289/ehp.11235

**Published:** 2008-07

**Authors:** Gianpaolo Guzzi, Paolo D. Pigatto

**Affiliations:** Italian Association for Metals and Biocompatibility Research Milan, Italy E-mail: gianpaolo_guzzi@fastwebnet.it; Department of Technology for Health Dermatological Clinic, IRCCS Galeazzi Hospital, University of Milan, Milan, Italy

Woods et al. (2007) reported on exposure to dental amalgam fillings and urinary mercury excretion in children. They stated that “urinary mercury concentrations are widely used as a measure of mercury exposure from dental amalgam fillings.” We would like to point out some caveats about interpreting the results of mercury in urine.

Clarkson and Magos (2006) and others (Mutter et al. 2007; Nilsson and Nilsson 1986; Nuttal 2004) noted that urinary mercury is a rough indicator of mercury from dental amalgams. In fact, the urinary mercury concentration is unlikely to be a robust biological indicator for prolonged exposure to mercury vapor from dental amalgam. Previous postmortem studies in humans have shown that mercury levels originating from dental amalgam surfaces and retained in tissues are higher in brain regions and thyroid than those measured in renal cortex (Guzzi et al. 2006).

These findings are consistent with the fact that kidneys are the major contributors of urinary mercury (Magos and Clarkson 2006; Nuttal 2004), and the concentrations of mercury in urine may not reflect the tissue retention of mercury in more sensitive tissues such as brain and endocrine glands. This might explain the association between an increased frequency and severity of clinical symptoms among individuals with dental amalgams and consistently reduced levels of excretion of total mercury in urine (Minoia et al. 2006; Nilsson and Nilsson 1986).

In addition, Woods et al. (2007) listed several factors that may be involved in the differences in urinary mercury concentrations between the sexes. However, they did not mention bruxism in the text. Bruxism has an important causative role in the increased concentration of mercury in urine (Barregard et al. 1995). Because various reports have suggested that bruxing behavior may increase the urinary levels of mercury (Isacsson et al. 1997), Woods et al. should have included it as a potential confounder factor.

As a result of their randomized trials, Woods et al. (2007) evaluated the influence of sex on mercury excretion rates. They found that girls have a more significant increase in the rate of mercury excreted in urine than boys. Thus, this association might confer a lower mercury toxicity risks in girls.

Our experience regarding the care and treatment of adverse mercury amalgam events among adult individuals does not support the hypothesis that males might be more susceptible than females to the adverse events caused by long-term exposure to mercury vapor from amalgams (Guzzi et al. 2005). Our findings, which were derived from an ongoing study regarding clinically significant adverse events occurred in 289 adults patients due to mercury amalgam fillings, showed that females are two to three times more likely to develop local (e.g., lichenoid contact stomatitis) or systemic adverse health outcomes (e.g., skin disorders) compared with males [217 of 289 were women (75.09%) with a median age of 43; 72 of 289 were men (24.91%) with a median age of 40.5; female to male ratio, 3.1:1]. Therefore, in our experience, adult females were more likely to be affected by prolonged exposure to mercury vapor released from dental amalgams.

Moreover, given that inorganic mercury [Hg^2+^] binds mainly to thiol ligands [–SH] as homocysteine (Bridges and Zalups 2004), we suggest that future clinical trials addressing the role of sex in mercury excretion should include an evaluation of serum homocysteine, which is higher in males than in females and might account for an increased tissue retention of mercury (Novembrino et al. 2006).

Finally, Woods et al. (2007) did not consider the importance of determining whether the exposure to mercury vapor emitted from amalgams may affect the immune system of children (Pigatto and Meroni 2006). Indeed, mercury-induced immunotoxicity arises far earlier than overt toxicity in the renal and central nervous systems.

## Editor’s note

In accordance with journal policy, Woods et al. were asked whether they wanted to respond to this letter, but they chose not to do so.
